# Individual and Combined Effects of Nanoplastics and Cadmium on the Rhizosphere Bacterial Community of *Sedum alfredii* Hance

**DOI:** 10.3390/microorganisms12122471

**Published:** 2024-12-01

**Authors:** Yixiu Wang, Hongyan Cheng, Yuenan Li, Ruiyan Ning, Yonghui Lv, Qing Wang, Haibo Zhang, Na Liu

**Affiliations:** College of Resource and Environment, Shanxi Agricultural University, Taigu 030801, China; wangyixiu2022@163.com (Y.W.); ndchenghy@163.com (H.C.); liyuenan1202@163.com (Y.L.); ningry2023@163.com (R.N.); 15290536899@163.com (Y.L.); wangqing20240805@163.com (Q.W.); haiboz666@163.com (H.Z.)

**Keywords:** polystyrene nanoplastics, cadmium, combined pollution, bacterial community, metabolic pathways

## Abstract

Nanoplastics (NPs) and cadmium (Cd) coexist in soil, but the combined effects of NPs and Cd on the rhizosphere bacterial community remain unknown. In this study, high-throughput sequencing and PICRUSt2 functional analysis were employed to explore the individual and combined effects of polystyrene (PS) NPs (low concentration [N1, 100 mg·kg^−1^] and high concentration [N2, 1000 mg·kg^−1^]) and Cd (low concentration [C1, 0.6 mg·kg^−1^] and high concentration [C2, 4 mg·kg^−1^]) on the diversity, structural composition, and function of the rhizosphere bacterial community associated with *Sedum alfredii* Hance. Individually, PS NPs and Cd significantly reduced the soil pH, while the combined treatments induced a more significant decrease in pH. In contrast, combined PS NPs and Cd significantly increased the diethylenetriaminepentaacetic acid–Cd (DTPA-Cd) and total Cd concentrations. Compared with individual treatments, C2N2 significantly increased DPTA-Cd by 4.08%. N1 had no significant effect on the Chao1, observed species, or Shannon indices, while N2 significantly reduced the richness and diversity of the rhizosphere bacteria and altered their community structure. Furthermore, adding PS NPs exacerbated the effect of Cd on rhizosphere bacterial communities. Compared with individual Cd treatments, C2N2 significantly reduced the relative abundances of Actinobacteriota, Bacteroidota, Crenarchaeota, and Myxococcota by 19.76%, 2.01%, 1.49%, and 2.00%, respectively, and significantly increased the relative abundance of Acidobacteriota by 16.05%. A cluster heat map showed that the combined treatments attenuated glycan biosynthesis and metabolic function and enhanced the metabolism of cofactors and vitamins. These findings illuminate rhizosphere processes under co-contamination with heavy metals and PS NPs, supporting the practical application of phytoremediation to alleviate combined Cd and PS NP pollution.

## 1. Introduction

China currently faces a serious plastic pollution problem. According to statistics, in 2020, China used 90,877 million tons of plastic, and the amount of plastic waste reached approximately 60 million tons, 32% of which entered the terrestrial environment [1]. Plastics decompose into smaller pieces through mechanical action, biodegradation, and photodegradation [2]. Microplastics (MPs) are generally defined as plastic particles less than 5 mm in size, and nanoplastics (NPs) are generally defined as plastic particles in the size range of 1 nm to 500 nm [3,4]. These MPs/NPs are widespread in the environment, including the air, water, and soil, owing to their high enrichment, low degradability, and easy migration, and they have been listed as new global environmental pollutants [5,6,7]. It is estimated that terrestrial ecosystems receive 4 to 23 times more MPs/NPs than aquatic ecosystems [3,8,9]. Therefore, the influence of MPs/NPs on soil is worth further investigation.

Owing to their small particle size, MPs and NPs are easily transferred through the food chain. In addition, due to their specific surface area and functional groups, MPs/NPs easily adsorb environmental pollutants and can migrate to different environments as carriers of these pollutants [10]. MPs/NPs do not exist alone in the environment, so their co-existence with other pollutants is inevitable. Therefore, the negative environmental effects caused by the combined pollution of MPs/NPs and other environmental pollutants should not be underestimated.

According to the Bulletin of the National Soil Pollution Survey, cadmium (Cd) ranked first among the inorganic pollutants in China, and over 7.0% of Cd-contaminated areas exceeded the standard for Cd pollution [11]. Even trace concentrations of Cd can affect the physiological and biochemical processes of plants and biological functions in soil. They can even accumulate through the food chain to affect human health, for example, resulting in “itai-itai” disease in Japan [12,13,14]. The combined effects of MPs/NPs and Cd may differ from those caused by individual pollutants. For example, previous research showed that polyethylene (PE) and polylactic acid (PLA) MPs increased the concentration of diethylenetriaminepentaacetic acid–cadmium (DTPA-Cd) in soil, and when present together, PE MPs-Cd and PLA MPs-Cd affected the relative abundance of arbuscular mycorrhizal fungi (AMF) [15]. MPs/NPs can alter the texture and structure of soil and affect soil fauna, plant growth, and microbial activity, but their effects are highly dependent on their size, type, and concentration [16,17,18]. Polystyrene (PS) is a common plastic in the soil [19]. However, little is known regarding the combined contamination of PS NPs and heavy metals, especially Cd, and whether the co-existence of PS NPs and Cd can affect soil physicochemical properties and microbiota remains unclear.

Phytoremediation is an emerging green technique in which plants are used to remove environmental pollutants. Compared with other heavy metal removal methods, phytoremediation has good flexibility, improved cost-effectiveness, and high acceptance, and does not cause secondary pollution [20,21,22]. Phytoremediation is also a promising approach to MP removal [23]. Chemicals released by plant roots affect soil physicochemical properties and rhizosphere microbial activity, which in turn affect plant–microbe system interactions. *Sedum alfredii*, a native Chinese plant, is a Zn/Cd co-hyperaccumulator discovered in a Pb/Zn-rich area [24,25,26]. *S. alfredii* was found to grow in soil highly contaminated with Cd, Zn, Pb, and Cu, with no noticeable alteration in its microbial community or symptoms of phytotoxicity [27]. At present, much research in this area focuses on the effects of one or more heavy metals on the growth and rhizosphere microbial activity of *S. alfredii* [28,29]. However, little information is available regarding whether MPs/NPs, a new type of environmental pollutant, affect rhizosphere microbial activity in Cd-contaminated soil, so further research is needed.

In the present study, *S. alfredii* was taken as the test plant; the individual and combined effects of PS NPs and Cd on soil physical and chemical properties were analyzed; the rhizosphere bacterial community structure and composition of *S. alfredii* were investigated using high-throughput sequencing technology; and changes in soil function combined with PICRUSt2 functions were analyzed. Our previous research revealed that combined PS NP and Cd treatments exhibited stronger growth inhibition and toxicity on *S. alfredii* compared with single treatments [30]. The current study will further explain this phenomenon from the perspectives of soil physicochemical properties and rhizosphere microorganisms. The objectives of this study are as follows: (i) to assess the individual and combined effects of PS NPs and Cd on soil physicochemical properties (pH, DTPA-Cd, and total Cd) and (ii) to investigate the individual and combined effects of PS NPs and Cd on the diversity, structural composition, and function of the rhizosphere bacterial community. This study aimed to establish a theoretical foundation for the effect of combined PS NPs and Cd on rhizosphere bacterial communities, as well as practical guidance for restoring NP- and heavy-metal-contaminated farmlands.

## 2. Materials and Methods

### 2.1. S. alfredii Hance

*S. alfredii*, a Cd hyperaccumulator, was obtained from Taizhou City, Zhejiang Province, China. *S. alfredii* plants with uniform size and optimal growth were selected for a subsequent pot experiment.

### 2.2. Soil

Farmland soil that was not covered by agricultural plastic mulch was collected from the experimental station at the College of Resources and Environment of Shanxi Agricultural University (112°58′ E, 37°42′ N), Shanxi Province, China. After natural air drying, the soil was screened with a 2.0 mm sieve, thoroughly mixed, and stored for use. The soil was calcareous brown soil, and its original physical and chemical properties are presented in Table S1.

### 2.3. PS NPs

PS NPs with a particle size of 100~500 nm were purchased from Denka (Denka Inc., Tokyo, Japan). The structure of the PS NPs was observed using a scanning electron microscope (JEOL Ltd., Tokyo, Japan). Fourier transform infrared spectroscopy (FT-IR) (Thermo Fisher Scientific, Waltham, MA, USA) analysis was performed under a 400~4000 cm^−1^ spectrum, the number of scans was 32, and the photo resolution was 4 cm^−1^. The Brunauer–Emmett–Teller (BET) surface area of the PS NPs was measured using an automatic surface area analyzer (Micromeritics, Norcross, GA, USA) with N_2_ as the adsorbate at 77.30 K. Approximately 0.05 g of each sample was used, with a relative pressure (P/P°) range from 0.01 to 0.40 for the calculation. Measurements were repeated three times for consistency. The X-ray diffraction (XRD) of the PS NPs was measured using an X-ray diffractometer (Bruker Corporation, Karlsruhe, Germany).

### 2.4. Experimental Design

The concentrations of the PS NPs (100, 1000 mg·kg^−1^) were set to simulate NP contamination in agricultural soils based on reported contamination levels for MPs/NPs, ranging from 20 mg·kg^−1^ in undisturbed soil to 70,000 mg·kg^−1^ in the surrounding soil of industrial areas [31,32], as well as the relevant literature [33,34,35]. The Cd concentrations were set based on the average Cd level (ranging from 0.003 to 9.570 mg·kg^−1^, with an average of 0.6 mg·kg^−1^) and risk intervention value (4.0 mg·kg^−1^) for Chinese soil contamination in agricultural land [36,37]. Therefore, nine treatments were designed (Table 1). Each treatment had three replicates. Different concentrations of PS NPs and Cd solutions (analytical purity, Cd (NO_3_)_2_·4H_2_O) were added to the soil, and each plastic basin (22.8 cm × 17 cm × 17.5 cm) contained 3.5 kg soil. To ensure the accuracy of the experiment, the potted soil was first acclimatized for two weeks, during which complete interaction between the soil microbiota, soil, and PS NPs was allowed. Then, screened *S. alfredii* samples were transferred into the acclimatized soil, with one plant per pot. This experiment was cultivated in a greenhouse with a day–night (12 h/12 h) temperature range of 28~23 °C, a light intensity of 15,000 lux, and a relative humidity range of 50~65%. Before planting, compound fertilizer (N-P_2_O_5_-K_2_O, 29-13-9) was used as a basal fertilizer. *S. alfredii* was harvested after six months.

### 2.5. Rhizosphere Soil Collection

Rhizosphere samples were collected after *S. alfredii* was harvested. The plant was gently shaken to remove any loosely bound soil from the root system, and soil tightly bound to the root system (0~2 mm from the surface) was brushed off and collected as a rhizosphere soil sample. Rhizosphere soil samples were stored at −80 °C for later DNA extraction.

### 2.6. Analysis of Soil Physical and Chemical Properties

The soil physical and chemical indicators were determined using the process described by Shi et al. (1981) [38]. The pH (soil–water, 1:2.5, *w*:*v*) was measured using a pH meter (PH3-3C, Inesa Scientific Instrument Co., Ltd., Shanghai, China). The available Cd was extracted with diethylenetriaminepentaacetic acid (DTPA) solution and measured using an inductively coupled plasma emission spectroscopy instrument (Optima5300DV, PerkinElmer Inc., Wellesley, MA, USA). The soil total Cd (HNO_3_: HCl: HF, 1:1:2, *v*:*v*:*v*) was measured using an atomic absorption spectrometer (pinAAcle900Z, PerkinElmer Inc., Wellesley, MA, USA).

### 2.7. High-Throughput Analysis of Soil Bacterial Community

A soil DNA extraction kit (FastDNA^®^ SPIN Kit for Soil, MP BiomedicalsTM, Santa Ana, CA, USA) was employed to extract the total DNA from the rhizosphere microorganisms of each treatment sample. The concentration and purity of DNA were detected using a Nanodrop 2000 UV–visible spectrophotometer (ThermoScientific, Wilmington, DE, USA). The quality of extracted DNA was detected via 1% agarose gel electrophoresis. To achieve the correct concentration, DNA was diluted to 1 ng·µL^−1^ using sterile water. Qualified samples were sent to Beijing Nohe Technology Co., Ltd. (Beijing, China) for testing. The template was the DNA parent sequence, and the specific primer sequences were F: CCTAYGGGRBGCASCAG and R: GGACTACNNGGGTATCTAAT. Polymerase chain reaction (PCR) amplification was conducted for the 16S V3–V4 region (515F and 806R). All PCR reactions were performed using 15 µL of Phusion^®^ High-Fidelity PCR Master Mix (New England Biolabs, Ipswich, MA, USA), 0.2 µM of both forward and reverse primers, and approximately 10 ng of template DNA. The thermal cycling protocol included an initial denaturation at 98 °C for one minute, followed by 30 cycles of denaturation at 98 °C for 10 s, annealing at 50 °C for 30 s, and extension at 72 °C for 30 s, with a final extension step at 72 °C for 5 min. The IlluminaNovaSeq (San Diego, CA, USA) sequencing platform was used for the double-terminal sequencing of the PCR products at Beijing Nowo Technology Co., Ltd. (Beijing, China), and 250 bp paired-end reads were generated. The original sequencing data were obtained, spliced, and filtered, and noise reduction was conducted. Species annotation and abundance analysis were then performed using the validated data to reveal the species composition of the sample.

### 2.8. Data Processing and Analysis

To investigate the species composition of each sample, the validated data of all samples were clustered using the DADA2 software (V1.24.0) to obtain amplicon sequence variants (ASVs) (equivalent to 100% similarity clustering of the operational taxonomic units [OTUs]), and the representative sequences of the ASVs were annotated with species. An α-diversity analysis was performed based on ASVs (abundance indices: Chao1 and OTUs; diversity indices: Shannon and Simpson), and bar charts were designed. The β-diversity distance matrix was calculated using QIIME2, and a principal coordinate analysis (PCoA) chart was created based on the unweighted UniFrac distance. Based on the species annotation results, the species in the top 10 phyla and classes in terms of relative abundance were selected, and a stacking histogram of the species’ relative abundances was designed. The annotation results were associated with the corresponding functional database, and PICRUSt2 was employed to annotate species between the feature sequences to be predicted and the existing phylogenetic pathways in the software. Heat maps were drawn to illustrate the functional differences among samples using color shades.

The data are expressed as the mean ± SD. SPSS23.0 was used to perform a one-way analysis of variance (ANOVA; Duncan) to determine any significant differences between the treatments, while a two-way ANOVA (Duncan) was used to analyze the interactions between PS NPs and Cd. All figures were produced using Origin 2018.

## 3. Results

### 3.1. Analysis of Physicochemical Properties of PS NP

**Figure 1:**
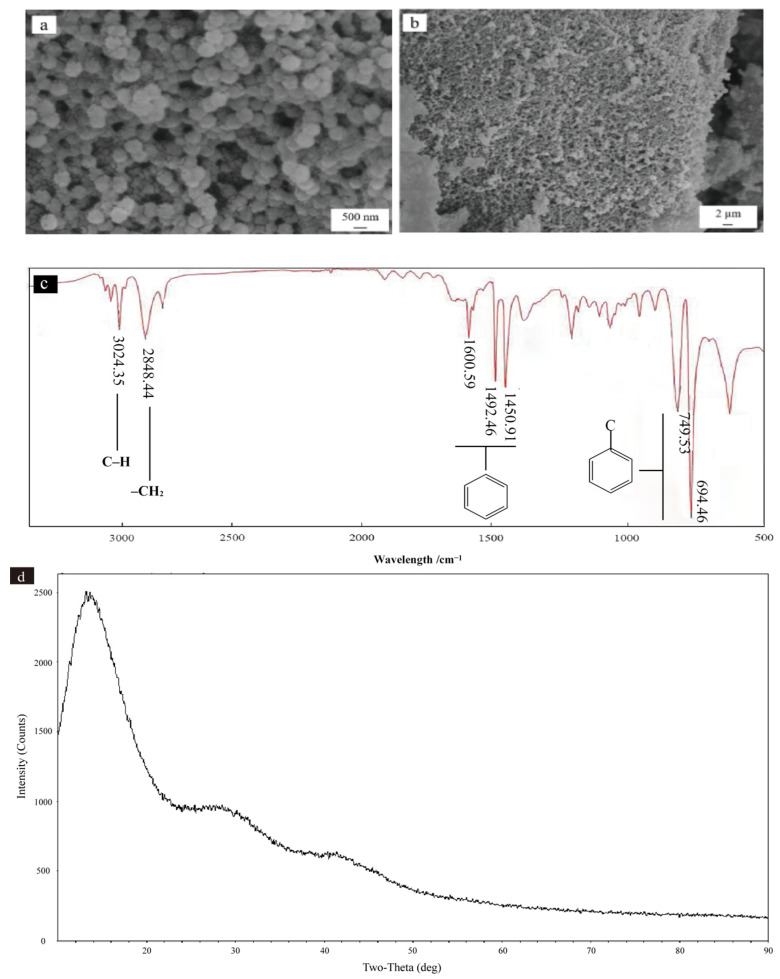
Nanoplastics’ characterization ((**a**,**b**): scanning electron micrographs of polystyrene nanoplastics; (**c**): Fourier transform infrared spectrum of polystyrene nanoplastics; (**d**): X-ray diffraction of polystyrene nanoplastics).

Figure 1a,b show that the PS NP microspheres were mainly in the form of small particle aggregates distributed in the system. The surfaces of the PS NPs were relatively homogeneous, accompanied by some fragments and micropores, and the particle size was 100~500 nm. FT-IR analysis showed that the PS NPs had benzene rings at 1600.59, 1492.46, and 1450.91 cm^−1^; an aromatic C-H bond at 3024.35 cm^−1^; and CH_2_ symmetric stretching vibration at 2848.44 cm^−1^. At wavelengths of 694.46 cm^−1^ and 749.53 cm^−1^, there was only one substituent on the benzene ring (Figure 1c). The BET surface area of the PS NPs was 22.7685 ± 0.3931 m^2^·g^−1^. The XRD spectra of the PS NPs are depicted in Figure 1d, showing that a broad and diffuse diffraction peak emerged in the low-angle region (10–30°), while the high-angle region (>40°) lacked distinct and sharp diffraction peaks.

### 3.2. Individual and Combined Effects of PS NP and Cd on Soil Physicochemical Properties

**Figure 2:**
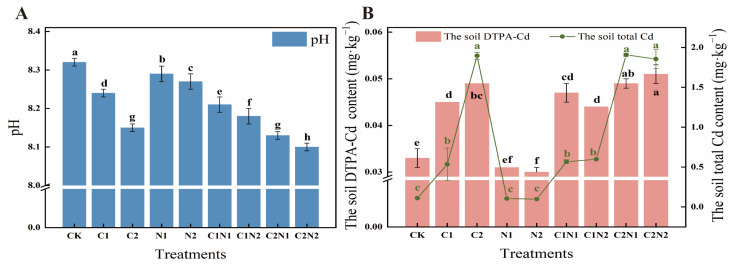
Effect of polystyrene (PS) nanoplastic (NP) and cadmium (Cd) on soil physical and chemical properties. (**A**) soil pH; (**B**) soil diethylenetriaminepentaacetic acid-cadmium (DTPA-Cd) and total Cd. Note: CK was the control treatment, with no added NPs or Cd. The C1 and C2 treatments had Cd concentrations of 0.6 mg·kg^−1^ and 4 mg·kg^−1^, respectively. The N1 and N2 treatments had NP concentrations of 100 mg·kg^−1^ and 1000 mg·kg^−1^, respectively. The C1N1, C1N2, C2N1, and C2N2 treatments were the combined NP and Cd treatments. Different lowercase letters indicate significant differences among treatments (*p* < 0.05). The error bar represents the standard error.

The changes in soil physical and chemical indices following exposure to individual and combined PS NP and Cd treatments at different concentrations were analyzed (Figure 2). Compared with the control (CK), pH and DTPA-Cd were significantly reduced under the individual Cd treatments and the combined PS NP and Cd treatments. Compared with CK, the high-concentration PS NP (N2) treatment decreased the pH from 8.32 to 8.27 (Figure 2A). Under the individual Cd treatments, the pH decreased with increased Cd concentrations, while the DTPA-Cd and total Cd increased compared with CK. Under the combined PS NP and Cd treatments, adding PS NPs increased Cd accumulation in the soil. Compared with the individual Cd treatment (C2), the high-concentration Cd and high-concentration PS NP (C2N2) treatment significantly increased the DTPA-Cd content by 4.08%. A significant interaction was found between PS NPs and Cd regarding DTPA-Cd (*p* < 0.05, Table S2).

### 3.3. Individual and Combined Effects of PS NP and Cd on Rhizosphere Bacterial Community Diversity

**Figure 3:**
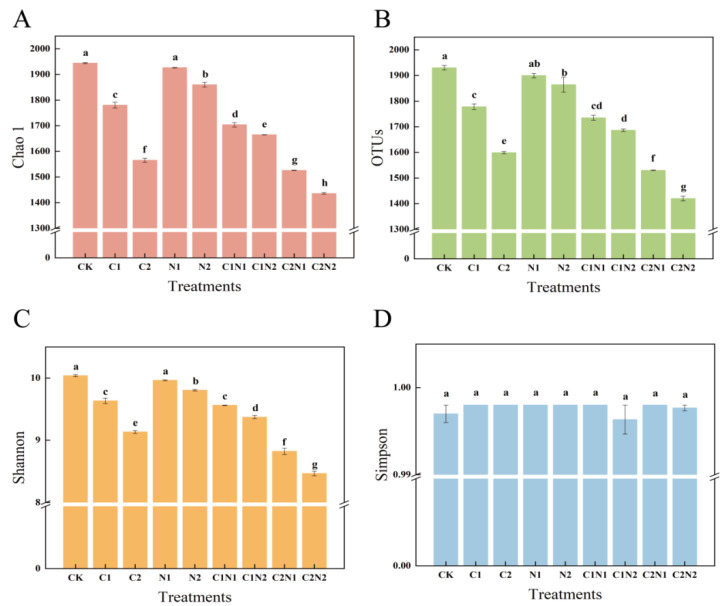
The α-diversity of the rhizosphere bacterial community under individual and combined polystyrene (PS) nanoplastic (NP) and cadmium (Cd) treatments: (**A**) Chao 1 index; (**B**) observed species (OTU) index; (**C**) Shannon index; (**D**) Simpson index. Note: CK was the control treatment, with no added NPs or Cd. The C1 and C2 treatments had Cd concentrations of 0.6 mg·kg^−1^ and 4 mg·kg^−1^, respectively. The N1 and N2 treatments had NP concentrations of 100 mg·kg^−1^ and 1000 mg·kg^−1^, respectively. The C1N1, C1N2, C2N1, and C2N2 treatments were the combined NP and Cd treatments. Different lowercase letters indicate significant differences among treatments (*p* < 0.05). The error bar represents the standard error.

This study analyzed the α-diversity of rhizosphere bacteria exposed to individual and combined treatments with PS NPs and Cd at different concentrations (Figure 3). The coverage index was >0.99, suggesting that the sequencing results provided an accurate representation of the sample. Compared with CK, the Chao1 and OTU indices of the PS NPs and Cd at different concentrations were significantly reduced. Compared with CK, the N1 treatment had no significant effect on the Chao1 and OTU indices, while the N2 treatment significantly reduced the Chao1 and OTU indices by 4.33% and 3.42%, respectively (*p* < 0.05, Figure 3A,B). In the individual Cd treatments, a higher Cd concentration resulted in a lower Chao1 index (the index values decreased by 8.42% and 18.75% for C1 and C2, respectively) and a lower OTU index (the index values decreased by 7.88% and 17.17% for C1 and C2, respectively) compared with CK. Among the combined treatments, the C2N2 treatment had the lowest Chao1 and OTU index values, with decreases of 9.09% and 11.18%, respectively, compared with the C2 treatment (*p* < 0.05, Figure 3A,B). The results demonstrated that adding PS NPs significantly reduced the abundance of rhizosphere bacteria and exacerbated the effects of Cd on the abundance of the rhizosphere bacterial community. A significant interaction was found between PS NPs and Cd regarding the Chao1 and OTU indices (*p* < 0.01, Table S2).

There was no significant difference in the Simpson index among all treatments (*p* > 0.05, Figure 3D). In the individual PS NP treatments, there was no significant difference in Shannon index values between CK and the addition of 100 mg·kg−1 PS NPs (N1) (*p* > 0.05, Figure 3C). Compared with CK, the N2 treatment significantly reduced the Shannon index by 2.33% (*p* < 0.05, Figure 3C). Therefore, the N1 treatment increased and maintained the diversity of the rhizosphere bacterial community, whereas high concentrations of PS NPs decreased the diversity of the rhizosphere bacterial community. The C1 and C2 treatments decreased the Shannon index by 4.02% and 9.01%, respectively, compared with CK, and the diversity of the rhizosphere bacterial community decreased at a higher Cd concentration. Compared with the individual Cd treatment (C2), the C2N2 treatment had low Shannon index values, decreasing by 7.29%, suggesting that adding PS NPs significantly reduced the diversity of the rhizosphere bacterial community. In addition, the influence of Cd on rhizosphere bacterial community diversity intensified. The ANOVA results showed that the PS NP and Cd interaction significantly impacted the Shannon index (*p* < 0.001, Table S2).

### 3.4. Individual and Combined Effects of PS NP and Cd on Rhizosphere Bacterial Community Structure

In this study, unweighted UniFrac distance data were used to calculate and present a dimensionality reduction graph. The horizontal and vertical coordinates explain the variability in the data, with the PC1 and PC2 axes explaining 13.74% and 8.23% of the variability, respectively.

**Figure 4:**
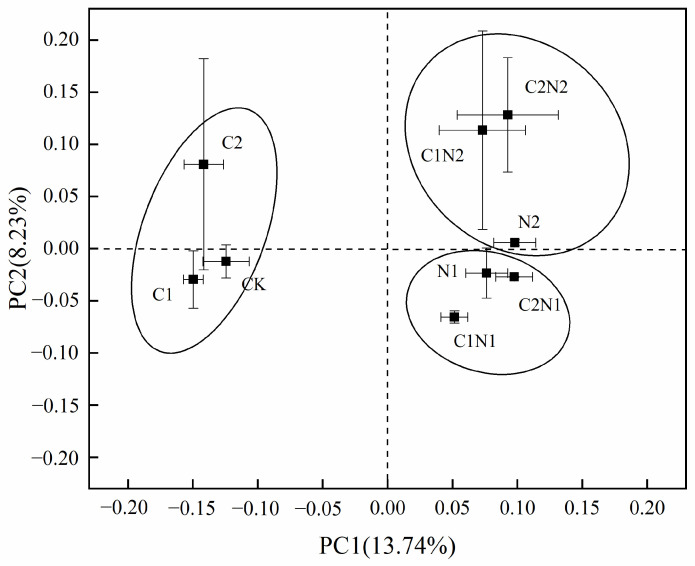
Principal coordinates analysis (PCoA) of rhizosphere bacterial community exposed to individual and combined polystyrene (PS) nanoplastic (NP) and cadmium (Cd) treatments. Note: CK was the control treatment, with no added NPs or Cd. The C1 and C2 treatments had Cd concentrations of 0.6 mg·kg^−1^ and 4 mg·kg^−1^, respectively. The N1 and N2 treatments had NP concentrations of 100 mg·kg^−1^ and 1000 mg·kg^−1^, respectively. The C1N1, C1N2, C2N1, and C2N2 treatments were the combined NP and Cd treatments.

In Figure 4, the CK, C1, and C2 treatments are grouped on the left side of the first principal coordinate (PC1). This suggests that there was little difference in the composition of the rhizosphere bacterial community between the individual Cd treatments and CK, and that there was high similarity in the bacterial community structure even under the high-concentration Cd treatment. All PS NP treatments are grouped on the right side of Figure 4, indicating that adding PS NPs changed the composition of the rhizosphere bacterial community compared with CK.

The CK, N1, C1N1, and C2N1 treatments (with PS NP concentrations of 100 mg·kg^−1^) are grouped at the bottom of Figure 4 according to the second principal coordinate (PC2), while the N2, C1N2, and C2N2 treatments (with PS NP concentrations of 1000 mg·kg^−1^) are grouped at the top of the figure. This suggests that the rhizosphere bacterial community composition differed under a high concentration of PS NPs compared with a low concentration, further demonstrating that different concentrations affect rhizosphere bacterial community composition and structure.

### 3.5. Individual and Combined Effects of PS NP and Cd on Rhizosphere Bacterial Community Composition

#### 3.5.1. Analysis of Common and Unique Species

**Figure 5:**
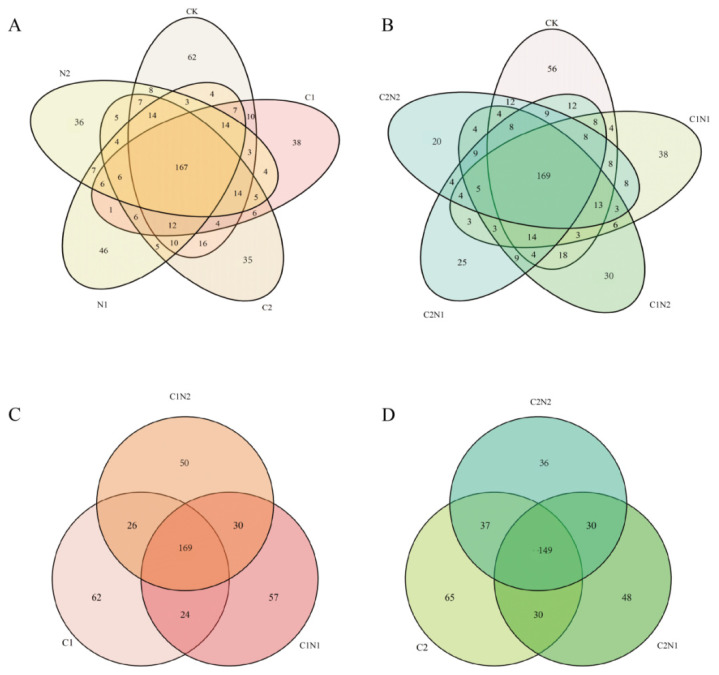
Venn diagram of the rhizosphere bacterial community exposed to individual and combined polystyrene (PS) nanoplastic (NP) and cadmium (Cd) treatments. The number of OTUs common and unique to (**A**) CK and individual PS NP and Cd treatments; (**B**) CK and combined PS NP and Cd treatments; (**C**) lower-Cd-concentration treatment and combined PS NP and lower-Cd treatment; (**D**) higher-Cd-concentration treatment and combined PS NP and higher-Cd treatment. Note: CK was the control treatment, with no added NPs or Cd. The C1 and C2 treatments had Cd concentrations of 0.6 mg·kg^−1^ and 4 mg·kg^−1^, respectively. The N1 and N2 treatments had NP concentrations of 100 mg·kg^−1^ and 1000 mg·kg^−1^, respectively. The C1N1, C1N2, C2N1, and C2N2 treatments were the combined NP and Cd treatments.

Figure 5A shows that the highest number of unique OTUs (62) was found in the CK rhizosphere samples. The diversity of the rhizosphere bacterial community was the highest when no exogenous pollutants were added. In the individual PS NP treatments, the numbers of unique OTUs decreased with an increase in the concentration of PS NPs, suggesting that the concentration of PS NPs was one of the factors affecting the bacterial diversity.

For CK and the combined PS NP and Cd treatments (Figure 5B), the total number of OTUs was 169, and there were fewer unique OTUs in the combined PS NP and Cd treatments than in CK. The C2N2 treatment had the fewest unique OTUs of all the rhizosphere samples, at only 20, suggesting that this combined treatment had the greatest effect on microbial diversity.

The total number of OTUs in the C1, C1N1, and C1N2 treatments was 169, and the total number of OTUs in the C2, C2N1, and C2N2 treatments was 149 (Figure 5C,D). With an increased concentration of PS NPs, the number of unique OTUs showed a decreasing trend from C1N1 to C1N2 and from C2N1 to C2N2 and was lower than under the C1 treatment. The same trend was observed when the Cd concentration was 4 mg·kg^−1^. These results suggest that the addition of PS NPs can aggravate the effect of Cd on microbial diversity.

#### 3.5.2. Individual and Combined Effects of PS NP and Cd on the Phylum-Level Composition of the Soil Bacterial Community

**Figure 6:**
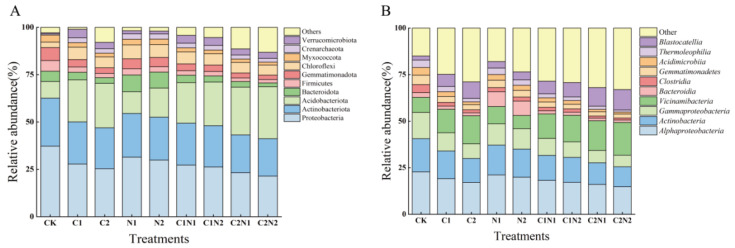
Composition of soil bacterial community induced by individual and combined polystyrene (PS) nanoplastic (NP) and cadmium (Cd) treatments: (**A**) phylum level; (**B**) class level. Note: CK was the control treatment, with no added NPs or Cd. The C1 and C2 treatments had Cd concentrations of 0.6 mg·kg^−1^ and 4 mg·kg^−1^, respectively. The N1 and N2 treatments had NP concentrations of 100 mg·kg^−1^ and 1000 mg·kg^−1^, respectively. The C1N1, C1N2, C2N1, and C2N2 treatments were the combined NP and Cd treatments.

The dominant bacterial species remained the same in all treatments, but the relative abundance of each species was altered. Figure 6A shows that the rhizosphere soil bacterial community mainly consisted of Proteobacteria, Actinobacteriota, Acidobacteriota, Bacteroidota, Firmicutes, Gemmatimonadota, Chloroflexi, Myxococcota, Crenarchaeota, and Verrucomicrobiota. Among these phyla, Proteobacteria was dominant in all treatments. CK had the highest relative abundance of Proteobacteria, which comprised 37.63% of the total abundance. The lowest relative abundance of Proteobacteria (21.42%) was detected in the C2N2 treatment. The relative abundances of Actinobacteriota and Acidobacteriota ranged from 19.76% to 25.56% and from 8.86% to 27.41%, respectively.

The relative abundances of Proteobacteria, Firmicutes, Gemmatimonadota, and Myxococcota significantly decreased by 16.37%, 40.92%, 24.82%, and 21.22%, respectively, in the N1 treatment compared with CK. By contrast, the relative abundance of Acidobacteriota significantly increased from 8.86% to 15.54% in the N2 treatment compared with CK (*p* < 0.05, Figure 6A).

The relative abundances of Proteobacteria, Actinobacteriota, Bacteroidota, Firmicutes, Gemmatimonadota, and Myxococcota significantly decreased in the individual Cd treatments compared with CK. By contrast, the relative abundances of Acidobacteriota, Chloroflexi, Crenarchaeota, and Verrucomicrobiota significantly increased (*p* < 0.05, Figure 6A).

Under the combined PS NP and Cd treatments, the addition of PS NPs increased the influence of Cd on the phylum-level composition of the soil bacterial community compared with the individual Cd treatments. Compared with the C2 treatment, the C2N2 treatment significantly decreased the relative abundances of Actinobacteriota, Bacteroidota, Crenarchaeota, and Myxococcota by 19.76%, 2.01%, 1.49%, and 2.00%, respectively, while the relative abundance of Acidobacteriota significantly increased by 16.05% (*p* < 0.05, Figure 6A).

Interactive effects were observed between PS NPs and Cd regarding the phylum-level composition of the soil bacterial communities (Proteobacteria, Bacteroidota, Firmicutes, Gemmatimonadota, Chloroflexi, Myxococcota, Crenarchaeota, and Verrucomicrobiota) (*p* < 0.05, Table S2).

#### 3.5.3. Individual and Combined Effects of PS NP and Cd on the Class-Level Composition of the Soil Bacterial Community

Figure 6B shows the top 10 most abundant bacterial classes in the rhizosphere samples, of which *Alphaproteobacteria* was dominant in soil samples exposed to different treatments. The relative abundance of *Alphaproteobacteria* accounted for 15.70–22.98% of the total abundance. This was followed by *Actinobacteria*, *Gammaproteobacteria*, *Vicinamibacteria*, *Bacteroidia*, *Clostridia*, *Gemmatimonadetes*, *Acidimicrobiia*, *Thermoleophilia*, and *Blastocatellia*.

The composition of the bacterial community in different rhizosphere samples was similar. The relative abundances of *Alphaproteobacteria*, *Actinobacteria*, *Gammaproteobacteria*, *Clostridia*, *Gemmatimonadetes*, and *Acidimicrobiia* significantly decreased in the individual PS NP treatments compared with CK. The relative abundance of *Bacteroidia* was two times higher under the N1 and N2 treatments than CK (*p* < 0.05, Figure 6B).

Compared with CK, the relative abundances of *Alphaproteobacteria*, *Actinobacteria*, *Acidimicrobiia*, and *Thermoleophilia* significantly decreased with an increased Cd concentration in the C1 and C2 treatments. Conversely, the C2 treatment significantly increased the relative abundances of *Vicinamibacteria* and *Blastocatellia* by 18.56% and 33.79%, respectively, compared with CK (*p* < 0.05, Figure 6B).

Compared with C1, the C1N1 treatment significantly decreased the relative abundance of *Actinobacteria* by 9.51%. The C1N2 treatment significantly decreased the relative abundances of *Alphaproteobacteria*, *Actinobacteria*, and *Thermoleophilia* by 6.35%, 10.77%, and 30.54%, respectively, while the relative abundance of *Blastocatellia* significantly increased by 16.78% (*p* < 0.05, Figure 6B). Compared with the C2 treatment, the C2N1 treatment significantly decreased the relative abundances of *Alphaproteobacteria* and *Actinobacteria*, while the relative abundance of *Blastocatellia* significantly increased. The C2N2 treatment significantly decreased the relative abundances of *Alphaproteobacteria* and *Actinobacteria* and significantly increased the relative abundances of *Vicinamibacteria* and *Blastocatellia* by 19.60% and 26.29%, respectively, compared with the C2 treatment (*p* < 0.05, Figure 6B).

Interactive effects were observed between PS NPs and Cd regarding *Vicinamibacteria*, *Bacteroidia*, and *Clostridia* (*p* < 0.01, Table S2).

### 3.6. Functional Analysis of Rhizosphere Bacterial Community Exposed to Individual and Combined PS NP and Cd Treatments

**Figure 7:**
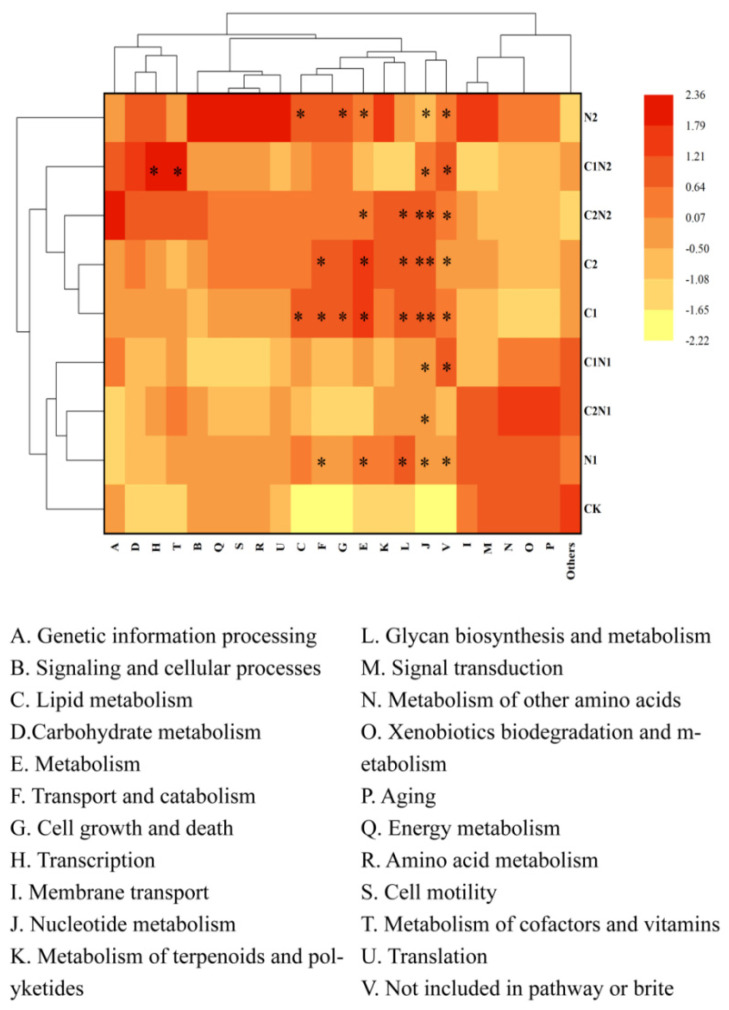
Comments on Kyoto Encyclopedia of Genes and Genomes (KEGG) functions of rhizosphere bacteria exposed to individual and combined polystyrene (PS) nanoplastic (NP) and cadmium (Cd) treatments. Note: CK was the control treatment, with no added NPs or Cd. The C1 and C2 treatments had Cd concentrations of 0.6 mg·kg^−1^ and 4 mg·kg^−1^, respectively. The N1 and N2 treatments had NP concentrations of 100 mg·kg^−1^ and 1000 mg·kg^−1^, respectively. The C1N1, C1N2, C2N1, and C2N2 treatments were the combined NP and Cd treatments. Significance levels: * *p* < 0.05, ** *p* < 0.01.

Figure 7 shows eight primary functional biological metabolic pathways in the Kyoto Encyclopedia of Genes and Genomes (KEGG) database. In this experiment, seven primary functional biological metabolic pathways were annotated. These consisted of genetic information processing, metabolism, cellular processes, environmental information processing, BRITE hierarchies, organismal systems, and not included in a pathway or BRITE. The relative abundance of the BRITE hierarchies was higher than that of the other six functions. The 7 primary functional biological metabolic pathways can be subdivided into 22 secondary functional biological metabolic pathways. Signaling and cellular processes had the highest relative abundance in all treatment groups, accounting for 4.51%~4.65% of the total abundance, while translation had the lowest relative abundance of the total at 0.14%~0.15%. The relatively abundant functions consisted of cell growth and death, genetic information processing, and membrane transport, accounting for 0.93%~1.00%, 0.84%~0.86%, and 0.59%~0.64% of the total abundance, respectively.

The functional differences between the effects of the individual and combined PS NP and Cd treatments on rhizosphere bacteria were analyzed. The results showed that PS NPs significantly enhanced metabolism and nucleotide metabolism compared with CK (*p* < 0.05, Figure 7). In the individual Cd treatments, compared with CK, metabolism, transport and catabolism, nucleotide metabolism, glycan biosynthesis and metabolism, and other functions were significantly different (*p* < 0.05, Figure 7). The difference in nucleotide metabolism was extremely significant in the individual Cd treatments compared with CK (*p* < 0.01, Figure 7).

Metabolism, glycan biosynthesis and metabolism, and the metabolism of cofactors and vitamins were significantly different in the combined PS NP and Cd treatments compared with the individual Cd treatments. The metabolism function was significantly weaker under the C2N1 treatment than the C2 treatment. Glycan biosynthesis and metabolism and cofactor and vitamin metabolism were significantly different in the C1N2 treatment compared with the C1 treatment, with weakened glycan biosynthesis and metabolism and enhanced cofactor and vitamin metabolism in the C1N2 treatment (*p* < 0.05, Figure 7).

Cluster analysis showed that the functions of the rhizosphere bacteria in the individual and combined PS NP and Cd treatments were mainly divided between two categories based on the concentration of PS NPs. One category consisted of the low-concentration PS NPs (N1, C1N1, and C2N1) and the other included the high-concentration PS NPs (C1N2 and C2N2). Notably, CK was grouped into the low-concentration PS NP category, while the individual Cd treatments (C1 and C2) were grouped with the high-concentration PS NP category, suggesting their functional similarities.

## 4. Discussion

The FT-IR analysis showed that the peaks of the test material were typical characteristic peaks of PS NPs [39] (Figure 1c). The PS was an amorphous polymeric material, and the result of the broad peak in Figure 1d is consistent with the expected characteristics of this type of material. Furthermore, this broad peak showed a low degree of crystallinity; indeed, the nanoscale nature of PS may result in a reduced degree of crystallization [40]. Traditional NPs (such as PE, PS, and polypropylene [PP]) persist longer than biodegradable NPs (such as PLA, polyhydroxybutyrate [PHB], and polysuccinate [PBS]) in the environment [41]. PS NPs have an undeniably significant effect on soil physicochemical properties and bacterial communities.

pH plays a crucial role in plant growth and vital microbial activity [42]. In this study, the initial experimental soil was alkaline (pH = 8.32) (Table S1), which may have affected nitrogen cycling and organic matter decomposition in the soil [43]. The current study found that pH decreased with an increased PS NP concentration and showed a dose-dependent effect (Figure 2A). In agreement with this finding, Li et al. (2023) found that PE and PLA MPs decreased the pH of Cd-contaminated soil [44]. The same results were found by Hou (2020) [45]. There are several possible explanations for the decrease in pH caused by NPs: (1) The compounds released during the aging and degradation of NPs can affect the root exudates, which affect soil pH. Kim et al. (2010) confirmed this in previous research [46]. (2) NPs may decrease soil pH by altering cation exchange capacity (CEC). Wang et al. (2021) attributed the decrease in pH from exposure to polyethylene (PE) MPs to alterations in soil CEC [47]. (3) NPs interfere with the normal vital activities of microorganisms, affecting their abundance, diversity, and metabolism, and consequently influencing soil pH. Rong et al. (2021) demonstrated that low-density polyethylene (LDPE) MPs altered the abundance of ammonia-oxidizing bacteria and the nitrification process, further decreasing the pH as the process released hydrogen ions [48]. Therefore, different types and doses of NPs affect soil pH. Feng et al. (2022) demonstrated that low doses of MPs (PS, PE, PLA, and PBS) had no significant effect on pH; conversely, high doses of PE and PS reduced it, and high doses of PLA and PBS increased it [49].

The chemical behavior of HMs in soil is significantly influenced by soil pH [50]. pH has a critical impact on the availability of HMs in soil; the lower the pH, the higher the availability of HMs [51]. In the present study, changes in the DTPA-Cd concentration were associated with the addition of PS NPs. The DTPA-Cd increased with an increased concentration of PS NPs (Figure 2B). Wang et al. (2020a, 2020b) made a similar finding, showing that co-existing MPs increased the DTPA-Cd concentration in soil [16,52]. There was an increasing DTPA-Cd trend in high-dose NP treatments, while there was a slight decline in DTPA-Cd in low-dose treatments (Figure 2B). Furthermore, the concentration of total Cd was not significantly different between the high-dose and low-dose treatments (Figure 2B). Studies have shown that adding MPs can reduce the adsorption capacity of soil for Cd but increase the desorption capacity [53,54]. Interestingly, a meta-analysis showed that the addition of MPs did not significantly alter DTPA-Cd in crops or non-crops, suggesting that plant type may not be a major factor influencing Cd availability [55]. This may be why the DTPA-Cd in the soil in this study did not change significantly before or after planting.

The complexity and the stability of the microbial community structure are closely related to the soil physical and chemical properties and the microbial diversity index [41]. In this study, the total potassium (18.54 g·kg^−1^) was sufficient to support healthy plant growth and the organic matter (12.37 g·kg^−1^) was sufficient to provide an adequate source of nutrients for microbial activity (Table S2) [56]. In the current study, compared with the individual Cd treatments, the combined PS NP and Cd treatments at different concentrations reduced the Chao1, OTU, and Shannon indices of rhizosphere microorganisms associated with *S. alfredii* (Figure 3). In a previous study on wolfweed, adding MPs exacerbated the influence of Cd on the bacterial community’s structure and significantly reduced its diversity [57]. The higher the concentration of PS NPs, the lower the bacterial community diversity index (Figure 3). This was also demonstrated in Pb-Zn-contaminated soil in a previous work, in which MP addition reduced the bacterial α-diversity of Pb-Zn-contaminated soil, and the effect was stronger with a higher dose of MPs (2%) [49]. In conclusion, adding PS NPs increased the influence of Cd on the soil microbial community and reduced its richness. The richness was limited by the concentrations of Cd and PS NPs, showing a dose–response effect. The PCoA showed that the CK, C1, and C2 treatments were far apart from the N1 and N2 treatments on both sides of the coordinate axis (Figure 4). This suggests that the bacterial community composition under the PS NP treatments significantly differed from that of the other treatments. This phenomenon can be attributed to a “regulatory film” that immediately forms on the surface of environments containing dissolved organic matter [58]. McCormick et al. (2014) found that NPs provide an ideal ecological niche for microorganisms owing to their specific surface area and strong hydrophobicity, promoting surface microbial colonization and biofilm formation [59]. Similarly, Rummel et al. (2017) demonstrated that microorganisms release extracellular enzymes that decompose polymers into monomers, changing the surface properties of NPs [60,61]. Furthermore, many studies have also revealed that the microbial community structure of the biofilm on the surface of NPs differs from that of the environment or medium [62,63,64]. In the current study, the composition of the rhizosphere bacterial community exposed to the N2 treatment was different from that exposed to the N1 treatment (Figure 4). The physical and chemical properties of NPs—such as their concentration, functional groups, hydrophobicity, roughness, crystallization degree, and melting temperature—affect microorganism colonization on their surfaces. In summary, the bacterial community structures of the Cd treatments and CK were significantly different from those of the PS NP treatments. Applying different concentrations of PS NPs also led to significant differences in the bacterial community structure.

NPs can affect the structure and composition of microorganisms by affecting the physical and chemical properties and nutrients of the soil. In this study, the initial experimental soil was alkaline (pH = 8.32) (Table S1), and the relative abundance of Acidobacteriota increased, possibly because the soil pH decreased after adding the PS NPs (Figure 6A). Xiong et al. (2018) found that the relative abundance of Acidobacteriota is negatively correlated with pH, and Acidobacteriota gene sequences have also been detected in neutral and alkaline environments [65]. Many studies have demonstrated that Proteobacteria and Actinobacteriota are the dominant bacteria in soil and sediment communities [66,67,68]. The conclusions of the present study are consistent with the aforementioned research (Figure 6A). In this work, the relative abundances of Proteobacteria, Firmicutes, Gemmatimonadota, and Myxococcota decreased after the introduction of PS NPs (Figure 6A), presumably owing to the release of chemical additives (such as colorants, plasticizers, and stabilizers) from the PS NPs in the environment, inhibiting the growth of these bacterial phyla [69,70]. Other reasons for the reduced relative abundances of these phyla may be that dominant bacteria had to compete for nutrients for growth and reproduction or that inferior bacteria could not obtain adequate nutrients [71]. In addition, many microorganisms from the *Proteobacteria* group are closely related to pHand decreases in pH may inhibit the lipase activity of *Proteobacteria* and, therefore, their growth [72]. The relative abundances of Bacteroidota, Chloroflexi, Crenarchaeota, and Verrucomicrobiota significantly increased after the addition of PS NPs (Figure 6A), possibly because PS NPs gradually decompose into small molecules under physical, chemical, and biological action, and the decomposed small molecules or polystyrene monomers can be absorbed by bacterial cells to support growth [66].

Heavy metal pollution adversely affects the original community structure and activity of microorganisms [70]. In this study, Proteobacteria was the dominant bacterial phylum in all treatments (Figure 6A). Proteobacteria can adapt to different soil conditions, such as common heavy metal pollution, antibiotics, and pesticides in soil. Drzewiecka et al. (2016) pointed out that these pollutants are likely to be used by Proteobacteria as sources of energy and nutrition [73]. The current study found that the combined application of PS NPs and Cd significantly reduced the diversity and abundance of the bacterial community compared with Cd alone (Figure 3), in addition to reducing the relative abundance of Proteobacteria (Figure 6A), which conforms with the results of Jiang et al. (2023) [68]. The relative abundance of Acidobacteriota was significantly higher under both individual and combined PS NP and Cd pollution than in CK (Figure 6A). Studies suggest that, in soil contaminated with heavy metals, an increase in the relative abundance of Acidobacteriota can help alleviate the toxic effects of heavy metals in various ways and improve the former’s tolerance for the latter [74,75]. The relative abundance of *Firmicutes* in soil was negatively correlated with the concentration of the heavy metal Cd (Figure 6A), consistent with changes in the relative abundance of *Firmicutes* observed in a previous study on ryegrass exposed to combined pollution [76]. The *Alphaproteobacteria* and *Gammaproteobacteria* classes belong to Proteobacteria and Vicinamibacteria, and *Blastocatellia* belong to Acidobacteriota. The changes in the relative abundances of these bacterial classes were consistent with the phyla changes (Figure 6B). Notably, *Blastocatellia* is widely present in soil contaminated with different heavy metals, and *Alphaproteobacteria* has been found to colonize PP-, PS-, and PE-contaminated soils [77]. In addition, the PS NPs added in the combined treatments could provide attachment points for both *Blastocatellia* and *Alphaproteobacteria*, forming an important part of the biofilm.

Changes in the soil bacterial community inevitably result in changes in bacterial function, and understanding the function of the bacterial community is beneficial to soil restoration and improvement [70]. The main metabolic pathways include amino acid metabolism, carbohydrate metabolism, and lipid metabolism, all of which are related to energy metabolism. Nucleotide metabolism, essential to physiological processes, is related to cell homeostasis, contributing to the production of ATP and GTP. The addition of PS NPs enhances metabolism and nucleotide metabolism (Figure 7), suggesting that rhizosphere microorganisms obtain the energy required for microbial cell activity by accelerating the metabolic process, resisting PS NP stress by enhancing their antioxidant capacity. The Cd treatments in this study enhanced the metabolism, transport and catabolism, nucleotide metabolism, and glycan biosynthesis and metabolism of the bacterial community (Figure 7). This enhancement of functions may represent the regulatory mechanism microorganisms use to adapt to Cd stress and reduce the impact of the resulting environmental stress [78].

## 5. Conclusions

This study demonstrated that the soil physical and chemical properties, rhizosphere bacterial community structure and composition, and function of the rhizosphere bacterial community were significantly influenced by individual and combined treatments with NPs and Cd. Compared with the individual Cd treatment, the combined treatments significantly reduced pH and the relative abundances of phyla, including *Proteobacteria* and *Bacteroidota*, and attenuated glycan biosynthesis and metabolic function. Furthermore, the combined treatments significantly increased the concentration of DTPA-Cd and the relative abundance of *Acidobacteriota* and enhanced the metabolism of cofactors and vitamins.

## Figures and Tables

**Table 1:** Experimental treatment.

Treatments	Cd (mg·kg^−1^)	PS NPs (mg·kg^−1^)
CK	0	0
C1	0.6	0
C2	4	0
N1	0	100
N2	0	1000
C1N1	0.6	100
C1N2	0.6	1000
C2N1	4	100
C2N2	4	1000

## Data Availability

The original contributions presented in the study are included in the article/Supplementary Material, further inquiries can be directed to the corresponding author/s.

## Supplementary Materials

The following supporting information can be downloaded at: https://www.mdpi.com/article/10.3390/microorganisms12122471/s1, Table S1: Original physical and chemical properties of soil. Table S2. Significance levels (*F* values) of PS NPs, Cd, and their interactions on measured variables, assessed by a two-way ANOVA.
