# Supplementary material for: Individual and Combined Effects of Nanoplastics and Cadmium on the Rhizosphere Bacterial Community of Sedum alfredii Hance
Source: Microorganisms. 2024 Dec 1;12(12):2471. doi: 10.3390/microorganisms12122471 (PMC11728214; doi:10.3390/microorganisms12122471)
Supplement: Supplementary file 1 [file microorganisms-12-02471-s001.zip › microorganisms-3293405-supplementary.pdf]

Supplementary Materials:

Individual and combined effects of nanoplastics and cadmium on rhizosphere bacterial community of *Sedum alfredii* Hance

Yixiu Wang, Hongyan Cheng, Yuenan Li, Ruiyan Ning, Yonghui Lv, Qing Wang, Haibo Zhang, Na Liu*

College of Resource and Environment, Shanxi Agricultural University, Taigu, 030801, China

* To whom correspondence should be addressed, E-mail: liuna@sxau.edu.cn, Tel: +86 155 3637 5845

Table S1 Original physical and chemical properties of soil

pH	Total Nitrogen (g·kg^{-1})	Alkaline Hydrolyzable Nitrogen (mg·kg^{-1})	Total Phosphorus (g·kg^{-1})	Fast-acting Phosphorus (mg·kg^{-1})	Total Potassium (g·kg^{-1})	Organic Matter (g·kg^{-1})	Total Cadmium (mg·kg^{-1})
8.32	0.38	47.98	0.52	6.62	18.54	12.37	0.09

Table S2 Significance levels (*F* values) of PS NPs, Cd, and their interactions on measured variables by a two-way ANOVA analysis

Variables		PS NPs	Cd	PS NPs × Cd
Soil physicochemical properties	pH	45.18 ***	421.98 *	0.21 ns
	DTPA-Cd	0.36 ns	422.62 ***	3.70 *
	Total Cd	0.07 ns	1187.04 ***	0.38 ns
Bacterial community diversity	Chao 1 index	221.03 ***	2914.74 ***	8.35 ***
	OTU index	61.78 ***	714.70 ***	5.90 **
	Shannon index	132.18 ***	1127.96 ***	17.02 ***
	Simpson index	0.77 ns	0.34 ns	1.11 ns
Phylum-level composition of the soil bacterial community	Proteobacteria	78.97 ***	363.91 ***	13.74 ***
	Actinobacteriota	7.27 **	22.67 ***	1.24 ns
	Acidobacteriota	7.01 ***	99.01 ***	1.59 ns
	Bacteroidota	11.24 ***	281.57 ***	20.99 ***
	Firmicutes	11.66 **	25.10 ***	4.18 *
	Gemmatimonadota	11.56 ***	100.98 ***	2.99 *
	Chloroflexi	14.42 ***	4.90 *	23.55 ***
	Myxococcota	10.07 **	69.59 ***	1.20 ns
	Crenarchaeota	19.04 ***	9.90 **	35.49 ***
	Verrucomicrobiota	0.86 ns	249.80 ***	6.08 **
Class-level composition of the soil bacterial community	*Alphaproteobacteria*	22.45 ***	108.88 ***	1.37 ns
	Actinobacteria	26.24 ***	121.24 ***	0.73 ns
	Gammaproteobacteria	5.34 *	32.67 ***	0.58 ns
	Vicinamibacteria	3.90 *	173.97 ***	5.39 **
	Bacteroidia	110.29 ***	810.34 ***	130.25 ***
	Clostridia	10.98 ***	23.98 ***	4.70 **
	Gemmatimonadetes	8.24 **	32.38 ***	0.95 ns
	Acidimicrobiia	11.19 ***	73.31 ***	2.26 ns
	Thermoleophilia	7.26 **	34.73 ***	0.27 ns
	Blastocatellia	41.67 ***	557.00 ***	2.12 ns

Significance levels: *$P < 0.05$, **$P < 0.01$, ***$P < 0.001$; ns non-significant effect
